# McGill University, Veterinary Department

**Published:** 1901-05

**Authors:** 


### COMMENCEMENT EXERCISES.

### McGILL UNIVERSITY, VETERINARY DEPARTMENT.

THE annual convocation for the conferring of degrees in Comparative Medicine and Veterinary Science at McGill was held in the old library, March 29th. Principal Peterson, as vice-chancellor of the university, presided, and there were also present Dr. Duncan McEachran, dean of the faculty of comparative medicine; Dean Johnson, Dean Bovey, Dean Walton, Dr. Baker, Dr. Charles McEachran, Dr. Girdwood, Dr. Adami, Prof. Penhollow, Prof. Wesley Mills, Prof. McBride, Prof. S. H. Capper, Dr. Charles H. Higgins, and Dr. Sugden. Mr. James W. Brackenridge acted as registrar.

The following have passed in the order given below:

*Veterinary Medicine and Surgery*, third year. Prof. D. McEachran; O. T. Amyrauld, J. T. Rork, D. S. Tamblyn.

*Cattle Pathology*, third year. Prof. Charles McEachran; D. S. Tamblyn, J. T. Rork, O. T. Amyrauld.

*Pathology*, third year. Prof. Adami; O. T. Amyrauld, George A. Kennedy, J. T. Rork, W. Manchester, D. S. Tamblyn.

*Materia Medica*, third year. Lecturer, B. A. Sugden, D.V.S.; George A. Kennedy, J. T. Rork, D. S. Tamblyn, W. Manchester, O. T. Amyrauld.

*Anatomy*, second year. Prof. M. C. Baker; A. D. Harrington, W. R. Blair, A. R. Douglas, S. Hadwen, W. H. Spear.

*Physiology*. Prof. Mills; A. D. Harrington, S. Hadwen, W. R. Blair, A. R. Douglas, W. H. Spear.

*Chemistry*. Prof. Girdwood; A. D. Harrington, S. Hadwen, A. R. Douglas, W. R. Blair, L. Doyle, A. S. Clark, W. H. Spear.

*Histology*, second year. Prof. Wilkins; S. Hadwen and W. R. Blair (equal), W. H. Spear, A. D. Harrington, L. Doyle, A. S. Clark.

*Histology*, first year. Prof. Wilkins; George Halcro, Hugh Gaw.

*Botany*. Prof. Penhollow; Hugh Gaw.

*Cynology*. Prof. Mills; S. Hadwen, W. R. Blair, A. R. Douglas, W. H. Spear, Hugh Gaw, A. D. Harrington, D. S. Tamblyn, J. T. Rork, A. S. Clark, O. T. Amyrauld, G. Halcro.

*Graduates of* 1901: O. T. Amyrauld, J. T. Rork, D. S. Tamblyn.

PRIZES. *Veterinary Medicine and Surgery.* Won by O. T. Amyrauld.

*Cattle Pathology.* Won by D. S. Tamblyn.

*Materia Medica.* Won by George A. Kennedy.

*Anatomy.* Won by A. D. Harrington.

*Physiology.* Won by A. D. Harrington.

*Chemistry.* Won by A. D. Harrington.

EXTRA PRIZES. For the best essay read before the Veterinary Medical Association. First, won by J. T. Rork; second, D. S. Tamblyn; third, O. T. Amyrauld.

Dr. Duncan McEachran gave a very able address, urging the increasing importance of the veterinary profession. The history of the South African war has shown the great value of the horse, and there is no knowledge upon which the world is so much dependent for its health and food supply as veterinary science. A disease like rinderpest can sweep a country of its cattle, and the more the diseases of animals are studied the less likely are such calamities to happen. The necessity of the proper equipment of veterinary colleges is more recognized year by year, and on the continent of Europe and in the United States no money is spared to provide suitable accommodation, equipment, and endowment for this science. He trusted that the university would soon be able to do more for the faculty of comparative medicine than it had in the past, and that the number of the students would soon reach the level of ten years ago. With some excellent practical advice to the graduating students, Dr. McEachran concluded.

Principal Peterson expressed the great debt of gratitude the university owed to the dean of the faculty for his work in the faculty of comparative medicine. He admitted that McGill had been rather a niggardly stepmother to this part of itself, but at any rate the veterinary college was a peculiarly happy family. Numbers are not everything, and it was a great thing that the faculty, unlike some of the comparative medicine colleges of other universities, was entirely free from debt. Addressing the new doctors he urged the necessity of the interest, self-culture, and the importance of the quiet sciences which underlie all medicine. He wished he could hold out the prospect of more help to the faculty from the university funds, but at present it was impossible, and he could only assure them of the university's sympathy. He gave them his very very best wishes for success in their chosen career, and was confident they would always be loyal to Old McGill.

